# Beyond the rare: a case of pseudomyogenic hemangioendothelioma treated sequentially with everolimus, denosumab, and pazopanib

**DOI:** 10.31744/einstein_journal/2024RC1107

**Published:** 2024-11-26

**Authors:** Douglas Dias e Silva, Camila Bobato Lara Gismondi, Murilo Marques Almeida Silva, Renee Zon Filipi, Fernando Moura, Reynaldo Jesus-Garcia, Roberto Carmagnani Pestana

**Affiliations:** 1 Hospital Israelita Albert Einstein São Paulo SP Brazil Hospital Israelita Albert Einstein, São Paulo, SP, Brazil.

**Keywords:** Hemangioendothelioma, Hemangioendothelioma, epithelioid, Neoplasms, vascular tissue, Everolimus, Denosumab, Pazopanib

## Abstract

Pseudomyogenic hemangioendothelioma is an ultra-rare vascular sarcoma that most commonly affects young adults, with a male predominance. It is diagnosed using a combination of imaging studies, histopathological examinations, and immunohistochemical staining. Surgical excision is the mainstay of treatment for pseudomyogenic hemangioendothelioma, with the goal of achieving a wide local excision and reducing the risk of recurrence. The role of systemic therapies is not well established because of the rarity of pseudomyogenic hemangioendothelioma, uncertainty regarding its response to currently approved medications, and lack of randomized controlled trials. We describe the case of an 18-year-old male patient diagnosed with multifocal pseudomyogenic hemangioendothelioma of the left lower limb who was treated with everolimus in addition to denosumab, achieving a partial response that was consolidated with resection, radiofrequency ablation, and radiotherapy of multiple local lesions, achieving a long-lasting response. Following subsequent disease progression, the patient responded favorably to pazopanib, with no significant toxicities.

## INTRODUCTION

Pseudomyogenic hemangioendothelioma (PMH) is an ultrarare vascular sarcoma that typically affects young adults.^(1–3)^ Pseudomyogenic hemangioendothelioma most commonly presents as a locally aggressive disease, although its metastatic potential has been previously described.^(4,5)^ Treatment is mostly based on a surgical approach, because the benefits of systemic therapy or radiation are not well defined.^(6)^

We report a case of a patient presenting with multifocal PMH in multiple tissue planes of the left lower extremity treated successfully with surgical excision in addition to everolimus, denosumab, pazopanib, radiofrequency ablation, and radiation therapy.

## CASE REPORT

In February 2021, an 18-year-old male patient with no known comorbidities presented with pain in his left leg and knee during walking. He denied any history of trauma, and a physical examination revealed no signs of infection. Magnetic resonance imaging (MRI) of the left knee revealed focal areas of bone signal alteration in the distal femur and subcutaneous tissue. Initially, these findings were interpreted as nonspecific and likely unrelated to the patient's symptoms. A subsequent MRI performed due to persistent symptoms 2 months later demonstrated a significant increase in the dimensions of most of the focal bone lesions described in the previous study, located in the left femur, left tibia, and left fibula, as well as the appearance of new soft tissue masses in multiple tissue planes. Subsequently, a positron emission tomography (PET-CT) revealed fluorodeoxyglucose (FDG)-avidity in multiple small osteolytic lesions affecting the distal femur, patella, tibia, fibula, talus, and calcaneus, all within the left lower limb. These lesions were predominantly cortical, exhibiting periosteal and endosteal erosion, and were associated with soft tissue lesions in the muscular and subcutaneous planes.

**Figure 1:**
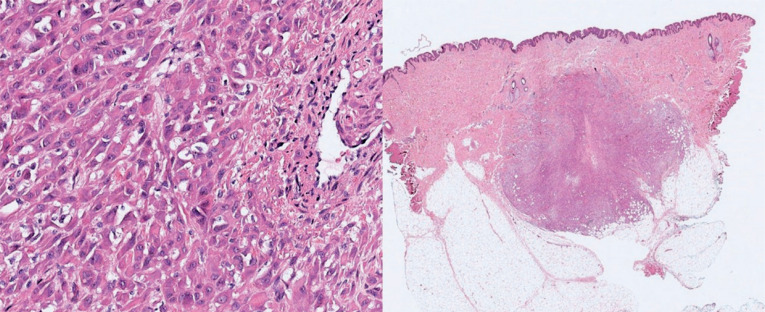
Pathology findings of pseudomyogenic hemangioendothelioma

In June 2021, excisional biopsies were performed on four samples: nodules in the subcutaneous tissue of the left thigh, nodules in the subcutaneous tissue of the left leg, left distal fibular lesions, and left calcaneal lesions. All four samples were processed for histopathological analysis (Figure 1) and immunohistochemistry (Table 1) to confirm the diagnosis of PMH.

**Table 1:** Immunohistochemistry analysis

Marker	Antibody/Clone	Result
CAMTA-1	Polyclonal	Negative
INI-1	MRQ-27	Preserved expression
Pan-cytokeratin	AE1/AE3	Positive
CD31	JC70A	Positive
ERG	EP111	Positive
CD34	QBEnd10mB	Negative
S100	Policlonal	Negative
Desmin	D33	Negative
Myogenin	F5D	Negative
Smooth muscle actin	1A4	Negative

In July 2021, monthly denosumab treatment was initiated at 120 mg. In addition, somatic genetic testing (Foundation Heme) was performed. After two doses of denosumab, the patient developed worsening symptoms and a new palpable soft tissue nodule. A new PET-CT scan showed an increase in the FDG uptake of bone lesions as well as in the number, dimensions, and FDG uptake of focal nodular lesions of the subcutaneous tissue/muscle of the left thigh and leg. At that time, the results of the somatic testing were available and showed no reportable genomic alterations (Table 2).

**Table 2:** Somatic panel

Foundation one heme
MSS, 0 mut/Mb, no reportable genomic alterations have been described
Variant of uncertain significance: TSC1 (K587R), MLH1 (Q689R), JAK1 (V464M), FBX011 (P49_Q50insP), ZNF217 (C697R)

**Figure 2:**
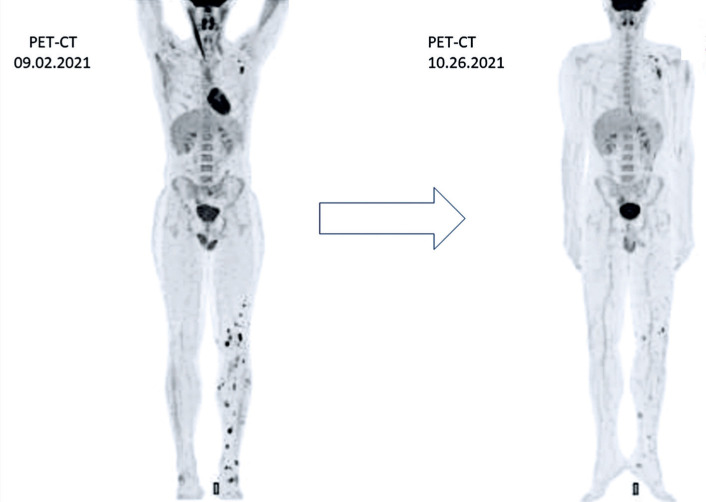
PET-CT showing response to everolimus. PET-CT, positron emission tomography-computed tomography

In August 2021, everolimus (10mg/day) was started in addition to the continuation of denosumab 120 mg/monthly due to the PET-CT findings of disease progression. Denosumab treatment was continued because of the presence of active bone lesions. In October 2022, a dose reduction to 5mg/day was required because of severe mucositis (grade 2 according to the Common Terminology Criteria for Adverse Events - CTCAE) refractory to topical symptomatic interventions. In the first everolimus response assessment, PET-CT revealed a partial response (Figure 2).

Therefore, everolimus (5mg/day) combined with denosumab (120mg monthly) was continued. In January 2022, a new PET-CT scan was performed, demonstrating a persistent partial response, although some areas exhibited a slight increase in FDG uptake. No evidence of new lesions was observed. The dose of everolimus was then increased, alternating between 5 and 10mg every other day, and treatment for mucositis was intensified. In April 2022, PET-CT showed that the majority of lesions maintained a partial response, although there were signs of increased glycolytic hypermetabolism in a small focal lesion in the soft tissues of the dorsum of the left midfoot, in the focal lesion in the projection of the soleus muscle of the middle or distal third of the left leg, and in most of the osteolytic lesions in the distal femur, tibia, and fibula on the left.

In May 2022, multiple soft tissue tumors of the left thigh, knee, leg, ankle, and foot were surgically resected. In addition, curettage was performed, followed by cementation of the left calcaneal lesion. Intraoperatively, the calcaneal tendon was evaluated, and tumor infiltration was suspected. After an onsite biopsy, the surgeon decided not to remove the entire calcaneal tendon. Moreover, radiofrequency ablation (Medtronics Osteocool™) of multiple lesions of the femur, tibia, fibula, ankle bones, and left foot was required. The pathology report confirmed PMH in all the samples. Most of the lesions had free margins. None of the specimens showed areas of necrosis, although there were occasional areas of fibrosis adjacent to the site of injury. A biopsy of the calcaneal tendon lesion confirmed PMH. Figure 2 shows the pathological findings.

In July 2022, magnetic resonance imaging revealed a slight increase in a single lesion located in the myotendinous transition of the calcaneus, which was previously untreated. Subsequently, the patient underwent radiotherapy treatment from 08/29/22 to 09/02/22 at a dose of 2500 cGy (divided into five sessions for the left calcaneal lesion and the same dose for the left fibula head). The patient was closely monitored with imaging examinations every 3 months and remained with no systemic treatment and no evidence of disease progression until May 2023.

**Figure 3:**
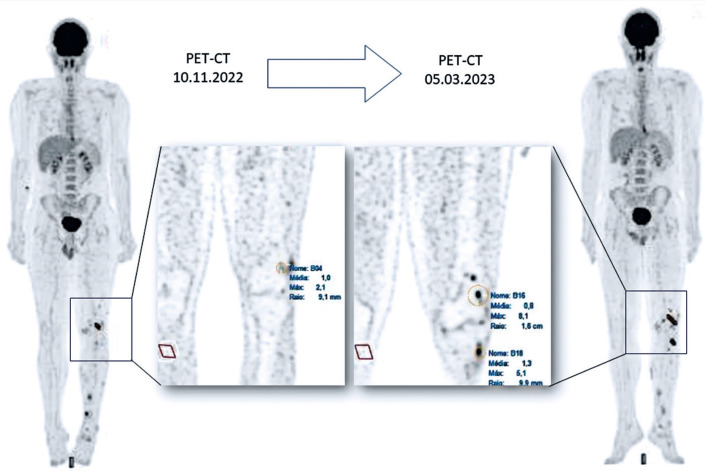
PET-CT showing progressive disease. PET-CT, positrone emission tomography-computed tomography

PET-CT performed in May 2023 showed progressive disease with the appearance of new uptake in lytic lesions in the left tarsal cuboid and posterior cortex of the left calcaneus and increased uptake in lytic lesions in the left patella, distal femur, and tibia (Figure 3).

At that time, owing to multifocal progression, we decided to pursue systemic therapy. Although the patient responded well to everolimus, mucositis significantly impaired his quality of life during therapy. Therefore, pazopanib treatment was initiated at a standard dose of 800mg/day. Immediately after the initiation of treatment, the patient was admitted to the hospital to treat the erysipelas of the left leg. Magnetic resonance imaging revealed soft tissue infection with no evidence of osteomyelitis.

**Figure 4:**
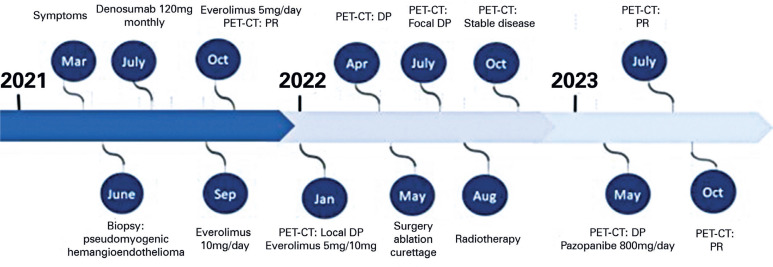
Timeline of diagnostic tests and therapy

PR: partial response; DP: disease progressive.

The first PET-CT scan after the start of pazopanib in July 2023 showed a reduction in the uptake of all bone lesions, which was considered a partial response in the femur, tibia, calcaneus, and talus. To date, pazopanib has shown no relevant toxicities. PET-CT performed in October 2023 confirmed an ongoing partial response to pazopanib. Figure 4 shows the timeline of all treatments received by the patient.

The case has been reported in line with the CARE guidelines for case reports.^(7)^

The study was approved by the Research Ethics Committee of *Hospital Israelita Albert Einstein* (CAAE: 77048623.6.0000.0071; #6, 647, 377).

## DISCUSSION

The diagnosis and management of ultra-rare sarcomas are challenging for pathologists, orthopedic surgeons, radiation oncologists, and medical oncologists.^(8–10)^ Pseudomyogenic hemangioendothelioma is an ultra-rare indolent vascular tumor initially described as "pseudomyogenic (fibroma-like) variant of epithelioid sarcoma" due to the lack of histologic evidence of vascular differentiation.^(11)^ Later, with additional immunohistochemical analyses, clinical recognition, and molecular diagnostics, this entity was better characterized.^(12)^

Pseudomyogenic hemangioendothelioma is commonly diagnosed in young adults, with a male predominance of 4–5:1, and a mean age of 30 years.^(13)^ Lower extremities are the most common sites of involvement (54%), as shown in this case report; however, other anatomic primary sites have been described.^(1)^ This clinical entity exhibits locally aggressive behavior in most cases. Moreover, multifocality is present in 66% of the cases, and bone involvement is reported in one-quarter of the patients. Multiple anatomical planes, such as the dermis, subcutis, and skeletal muscle, are involved in >90% of cases with multifocality, as was the case in our patient.^(14)^ The pattern of bone lesions is characterized by cortical and intramedullary lytic lesions with well-defined borders, without signs of local aggressiveness, and a low risk of metastatic disease.^(15)^

The diagnosis is challenging because of the rarity of this condition. In the largest reported series, the duration of symptoms before excision ranged from to 1-24 months (mean, 7 months). In the present case, the time from symptom onset to diagnosis was 4 months, which is in line with the literature. Potential differential diagnoses include vascular tumors, such as epithelioid hemangioendothelioma and epithelioid angiosarcoma, and non-vascular lesions, such as metastatic carcinoma, lymphoma, multiple myeloma, rhabdomyosarcoma, and epithelioid sarcoma. Consequently, a comprehensive diagnostic approach consistent with imaging (radiological, magnetic resonance imaging, and PET-CT), in addition to pathological findings, is relevant for a correct diagnosis.^(16)^

Histopathologically, the tumor was characterized by epithelioid-to-spindle endothelial cells with dense eosinophilic cytoplasm, few intracytoplasmic lumens, and no apparent vascular differentiation. These components present with mild-to-moderate cytological atypia, low mitotic activity, and admixed neutrophils. Immunophenotype patterns usually show strong positivity for keratin, Fli-1, and ERG, frequently positive for CD31 but negative for CD34; nuclear expression of SMARCB1 (INI-1) is retained.^(17)^ The t(7;19) (q22;q13) translocation has been described in most PMH cases, leading to a fusion between SERPINE1 and FOSB.^(18)^ In addition, other FOSB fusion partners have been described, such as ACTB,^(19)^ WWTR1,^(20)^ CLTC,^(21)^ EGFL7^(22)^ and POTE1.^(23,24)^

The recurrence pattern was mostly localized. Local recurrences and additional nodules in the same anatomic region have been reported in approximately 60% of patients with this diagnosis, usually within the first 2 years after the excision of the primary tumor.^(11)^ The standard treatment for PMH is local excision and/or curettage, although retrospective data have shown a significant percentage of amputations (20.7%).^(16)^ Notably, metastases are rare. In a large series of 50 patients, only one patient developed metastatic disease; however, long-term follow-up data are necessary to assess the frequency of late metastasis.^(1)^

Evidence of the efficacy of systemic therapy for PMH is limited. In recent case reports and small case series, chemotherapy, mammalian target of rapamycin (mTOR) inhibitors, anti-resorptive bone therapies, and tyrosine kinase inhibitors (TKI) have been investigated as treatment options, particularly for multifocal or recurrent lesions.^(25–29)^ Table 3 presents a literature review of case reports on the systemic treatment of PMH.

**Table 3:** Case reports of systemic therapy for pseudomyogenic hemangioendothelioma

Author	Details	Drug	Response	Biomarker
Joseph et al.^(25)^	Two patients, 45-year-old, right ilium PMH 22 year-old, right distal femur and left ilium	Gencitabine and docetaxel Everolimus after progression	1 PR 1 PD - on gencitabine and docetaxel PR to everolimus (second line)	All comers for gencitabine and docetaxel TSC1 mutation for second line everolimus
Ozeki et al.^(26)^	1 patient 15 years old multifocal disease in the proximal tibia and spinal metastasis	Everolimus	PR	
Gabor et al.^(30)^	One patient Nine years old Proximal metaphysis of the left femur and the pubic bone and further local infiltration	Chemotherapy (ifosfamide, actinomi-cyn, vincristina, carboplatina, epirrubicine, etoposídeo) Sirolimus	PD Stable disease in two years of follow-up	Positive for phospho-S6 protein and negative for VEGF expression
Danforth et al.^(31)^	One patient, 6-year-old	Sirolimus Zoledronic Acid	CR	
Brance et al.^(16)^	One patient, 25 years old Left femur, tibia, patella, ankle and foot	Pamidronate	CR	
Pranteda et al.^(27)^	One patient, 17 years old Multifocal on foot	Gemcitabine	CR	-
Otani et al.^(28)^	One patient, 20 years old Left lower extremity	Denosumab	PR	-
Pasricha et al.^(29)^	One patient, 23-year-old Multiple lesions on the right tibia and fibula	Denosumab	Stable disease	-
Al Hanash et al.^(32)^	One patient, 35-year-old, right tibia and foot	Pazopanib	Stable disease	-
van IJzendoorn et al.^(33)^	One patient, 17-year-old, multiple skin lesions on the head and neck	Docetaxel 1st line Telatinib 2nd line	PD CR	-
Wei et al.^(34)^	One patient, 51-year-old, recurrent soft tissue tumor combined with skin ulceration in her right calf after local resection	Adjuvant Everolimus	NED in 1 year	Mutation of the S-phase kinase-associated protein 2 gene (SKP2)

PR: Partial Response; PD: Progressive Disease; CR: Complete response; NED, no evidence of disease.

Everolimus is an mTOR inhibitor that has shown activity in various malignancies, including vascular sarcomas.^(35)^ Pseudomyogenic hemangioendothelioma, in particular, is linked to the distinct genetic rearrangement t(7;19) that leads to the formation of the SERPINE1-FOSB fusion gene. SERPINE1 encodes a protein within the serine protease inhibitor family, known as plasminogen activator inhibitor-1 (PAI-1). It has been documented that PAI-1 inhibits apoptosis by triggering the Akt pathway. Therefore, mTOR inhibitors are promising therapeutic targets in this setting.^(36,37)^ Ozeki et al. evaluated the efficacy of this agent in the treatment of PMH. A pediatric patient with high mTOR expression in PMH tissue showed a partial response to everolimus.^(26)^ Moreover, Joseph et al.^(25)^ treated a pediatric patient with everolimus and PMH, revealing a tuberous sclerosis 1(TSC1) mutation. TSC1 and TSC2 are tumor suppressors that inhibit Rheb, a small GTPase essential for mTOR activation.^(38)^

Joseph et al.^(25)^ reported the only case with an actionable mutation in DNA sequencing with a partial response to targeted treatment. Despite performing next-generation sequencing, we did not find any genomic alterations in our patient's tumor, and only variants of uncertain significance were identified. Therefore, the treatment the patient received was not targeted to any specific mutations but was based on previously reported drugs used for this disease.^(38)^

Denosumab is a monoclonal antibody that targets RANK ligands and has been approved for the treatment of osteoporosis, bone metastasis, and giant cell tumors.^(39)^ Otani et al. treated a single case of PMH of the bone with monthly denosumab administration, which resulted in symptomatic relief of ankle pain.^(28)^ Pasricha et al. used a six-weekly basis Denosumab protocol to achieve stable disease and symptomatic relief after 6 months.^(29)^

As our patient showed disease progression with denosumab and significant toxicity with everolimus, the second-line treatment was pazopanib. The efficacy of TKIs has been described in two previous case reports. The first study to use pazopanib was conducted by Alkanash et al. A 35-year-old male with PMH in his right lower limb was treated with first-line pazopanib and achieved a sustained partial response for 6 months after treatment initiation.^(32)^ Van Jzendoorne et al. described the case of a 17-year-old male who had multiple skin lesions on the head and neck, was diagnosed with PMH, and was treated with telatinib, a multi-tyrosine inhibitor. He had a complete response and continued to use the drug for 9 years when the treatment was stopped because of the unavailability of the drug. Four years after having stopped the medication discontinuation, the patient remained in complete response. In addition, this group created a model using normal endothelial cells, the most likely precursors of PMH, expressing the SERPINE1-FOSB fusion. This fusion product can control the expression and enhance PDGFRA and FLT1 levels. In this model, telatinib effectively inhibited the surface receptors FLT1, FLT4, and PDGFRA and disrupted the self-regulated expression of the fusion product. Consequently, telatinib and other TKIs can indirectly affect the expression of SERPINE1-FOSB and may serve as important therapeutic options for individuals with multifocal, unresectable PMH.^(33)^

## CONCLUSION

Further studies are required to determine the optimal dose, duration, and sequence of systemic therapies for pseudomyogenic hemangioendotheliomas. Here, we describe a case of sequential therapy with everolimus, denosumab, and pazopanib. Our report highlights that the use of systemic therapy in pseudomyogenic hemangioendothelioma represents a significant option for the management of this rare and challenging tumor, especially in cases where surgical resection is not feasible or has resulted in local recurrence or metastasis.
